# The pancreatic zymogen granule membrane protein, GP2, binds *Escherichia coli *type 1 Fimbriae

**DOI:** 10.1186/1471-230X-9-58

**Published:** 2009-07-23

**Authors:** Su Yu, Anson W Lowe

**Affiliations:** 1 Stanford University, Department of Medicine and the Stanford Digestive Disease Center, Stanford, USA

## Abstract

**Background:**

GP2 is the major membrane protein present in the pancreatic zymogen granule, and is cleaved and released into the pancreatic duct along with exocrine secretions. The function of GP2 is unknown. GP2's amino acid sequence is most similar to that of uromodulin, which is secreted by the kidney. Recent studies have demonstrated uromodulin binding to bacterial Type 1 fimbria. The fimbriae serve as adhesins to host receptors. The present study examines whether GP2 also shares similar binding properties to bacteria with Type 1 fimbria. Commensal and pathogenic bacteria, including E. coli and Salmonella, express type 1 fimbria.

**Methods:**

An *in vitro *binding assay was used to assay the binding of recombinant GP2 to defined strains of *E. coli *that differ in their expression of Type 1 fimbria or its subunit protein, FimH. Studies were also performed to determine whether GP2 binding is dependent on the presence of mannose residues, which is a known determinant for FimH binding.

**Results:**

GP2 binds *E. coli *that express Type 1 fimbria. Binding is dependent on GP2 glycosylation, and specifically the presence of mannose residues.

**Conclusion:**

GP2 binds to Type 1 fimbria, a bacterial adhesin that is commonly expressed by members of the *Enterobacteriacae *family.

## Background

GP2 is the major membrane protein in secretory granules of the exocrine pancreas [1-5]. Depending on the species, GP2's mass is 80–100 KDa and accounts for 35% of the total zymogen granule membrane protein [5]. The GP2 nucleotide sequence contains motifs consistent with domains for a signal sequence, DC8, EGF, ZP, and one specifying a glycosylphosphatidylinositol linkage to the membrane [6,1-9]. There are 7–10 potential asparagine-linked glycosylation sites depending on the species (10 in human GP2) [9]. During the secretory process, GP2 is cleaved from the membrane and secreted into the pancreatic duct along with the other digestive enzymes [1,8,10].

GP2's biologic function is unknown. Hypotheses proposed for GP2's function have included a role in pancreatic exocrine protein secretion; including the formation of secretory granules, or the packaging and sorting of digestive enzymes. An essential requirement for GP2, however, was not found when GP2 *null *mice harbored no detectable anomalies in either protein secretion or pancreatic acinar cell morphology [11].

The bacteria *Escherichia coli *belongs to the family *Enterobacteriacae *and is responsible for a large burden of human morbidity. Most commensal and pathogenic strains express Type I fimbria, which is a filamentous protein projection that serves as an adhesin to host receptors, and may be important for the colonization of specific niches [12,13]. An essential subunit of Type I fimbria is the FimH protein, which is located at the fimbrial tip. FimH mediates binding to mannose containing host receptors.

GP2's closest homologue is uromodulin, a protein expressed by the kidney that shows 52% identity and 67% conservation in amino acid sequence. Uromodulin is secreted into the urine and binds *E. coli *with Type 1 fimbriae. A role in host defense has been proposed in which uromodulin serves as a molecular decoy that prevents bacteria from binding to uroplakin, the host receptor in uroepithelia [14,15]. In addition, two independent laboratories have produced uromodulin *null *mice that showed increase sensitivity to urinary tract infections [14,16]. In this study, we demonstrate that GP2 binds *E. coli *that express Type 1 fimbria.

## Methods

### Reagents

Recombinant human GP2 protein was purified from culture supernatants of stably transfected Chinese Hamster Ovary cells as previously described[17]. In brief, the recombinant human GP2 was produced using the first 505 amino acids. The terminal 32 amino acids (a.a. 506–537), representing a hydrophobic domain that results in the formation of a glycosylphosphatidylinositol linkage to the membrane, was replaced by six histidine residues that enabled purification of the secreted protein with nickel-based affinity chromatography (Qiagen, Inc., Germantown, MD).

A rabbit anti-human GP2 polyclonal antibody was generated against amino acids 22–181 as previously described [18].

Uromodulin was purified from human urine as previously described [3,19]. Bovine serum albumin and methyl α-D-mannopyranoside were obtained from Sigma, Inc. (St. Louis, MO). Peptide:N-glycosidase F was obtained from New England BioLabs, Inc. (Ipswich, MA).

### Bacteria Strains, Culture, and Metabolic Labeling

Bacteria strains used in this study included AAEC185, AAEC185/pSH2, MG1655, AAEC072, KB91, NU14 and J96. The characteristics and sources for the bacteria are listed in Table 1. All strains were cultured in Luria-Bertani media and supplemented with ampicillin (50 μg/ml) or chloramphenicol (40 μg/ml) at 37°C as indicated.

**Table 1:** *E. coli *strains used in this study

Bacteria	Characteristics	Source
AAEC185 (-)	Derived from E. coli K-12 strain, MM294, in which the fim operon is deleted. Also recA^- ^[34].	Scott J. Hultgren, Washington Univ.
	
AAEC185/pSH2	The AAEC185 strain with the entire fim operon expressed from the pSH2 plasmid [35].	

AAEC072	Derived from E. coli K-12 strain, MG1655. Deletion of fimB-H [34,36].	Ian Blomfield, Univ. of Kent
	
MG1655	Wild type E. coli K-12 (Fim^+^, but most cells are phased off for fimbrial expression	

KB91	Derived from E. coli K-12 strain, MG1655, where the fim operon was deleted to produce AAEC191A [34]. The fimbrial M_1_L variant of FimH derived from intestinal E. coli was then expressed rom AAEC191A [25].	Evgeni V. Sokurenko, Univ. of Washington

Nu14	E. coli cystitis isolate that expresses the M_1_H variant of FimH [37].	Evgeni V. Sokurenko Univ. of Washington

J96	E. coli serotype O4:K6:45, a uropathogenic pyelonephritis isolate that expresses the M_1_H variant of FimH and P (PapG1 and G3) fimbriae.	Obtained from American Type Culture Collection (ATCC).

Bacteria were metabolically labeled with [^35^S]methionine as previously described [15]. Briefly, the bacteria were grown in Luria-Bertani medium at 37°C for 16 h, collected by centrifugation, and suspended in methionine- and cysteine-free Dulbecco's Modified Eagle's Medium (Mediatech, Inc., Manassas, VA) for 2 h at 37°C. [^35^S]methionine and [^35^S]cysteine (10 mCi/ml, MP Biomedicals, Solon, OH) was added for 2 h followed by 4 washes with PBS. The bacterial radioactive specific activity was determined by plating the bacteria on agar and determining the number of counts (cpm) per colony forming unit (CFU). The experimental data was expressed as CFUs. The radiolabeled bacteria were stored in 30% glycerol-PBS at -70°C.

### Bacterial Binding Assay

Ninety-six well polystyrene microtiter plates were incubated with 10 μg/ml of GP2, uromodulin, or BSA protein at room temperature for 30 min and then 4°C overnight. Unbound protein was removed by washing with PBS followed by a final incubation with 3% BSA in PBS to reduce non-specific binding. Radiolabeled bacteria were then reconstituted in PBS and 3% BSA, added to the well at 10^8 ^CFU/well, and incubated at room temperature for 2 h. Unbound bacteria were removed by washing with PBS. Bound bacteria were harvested with a solution of 1% SDS and quantified with a scintillation counter. All binding assays were performed in triplicate. Data for bacterial binding was expressed as CFU/well.

For assays that required deglycosylated GP2 or uromodulin, the protein was denatured in 0.5% SDS and 1% β-mercaptoethanol at 100°C for 10 min followed by incubation for 2 h in 0.05 M sodium phosphate buffer (pH 7.5), 2,500 units/ml P:Endoglycosidase F, 0.5% SDS, 1% β-mercaptoethanol and 1% NP-40 at 37°C.

Competitive inhibition of bacteria binding was studied by incubating with 1% (w/v) α-D-mannose or purified GP2 (50 μg/ml) in PBS for 2 h at 37°C.

### Statistics

Data are expressed as the mean ± standard deviation (SD). The student's *t *test was used to assess significance. A *p *< 0.05 was considered statistically significant.

## Results

### GP2 binds E. coli with FimH containing Type 1 fimbriae

**Figure 1:**
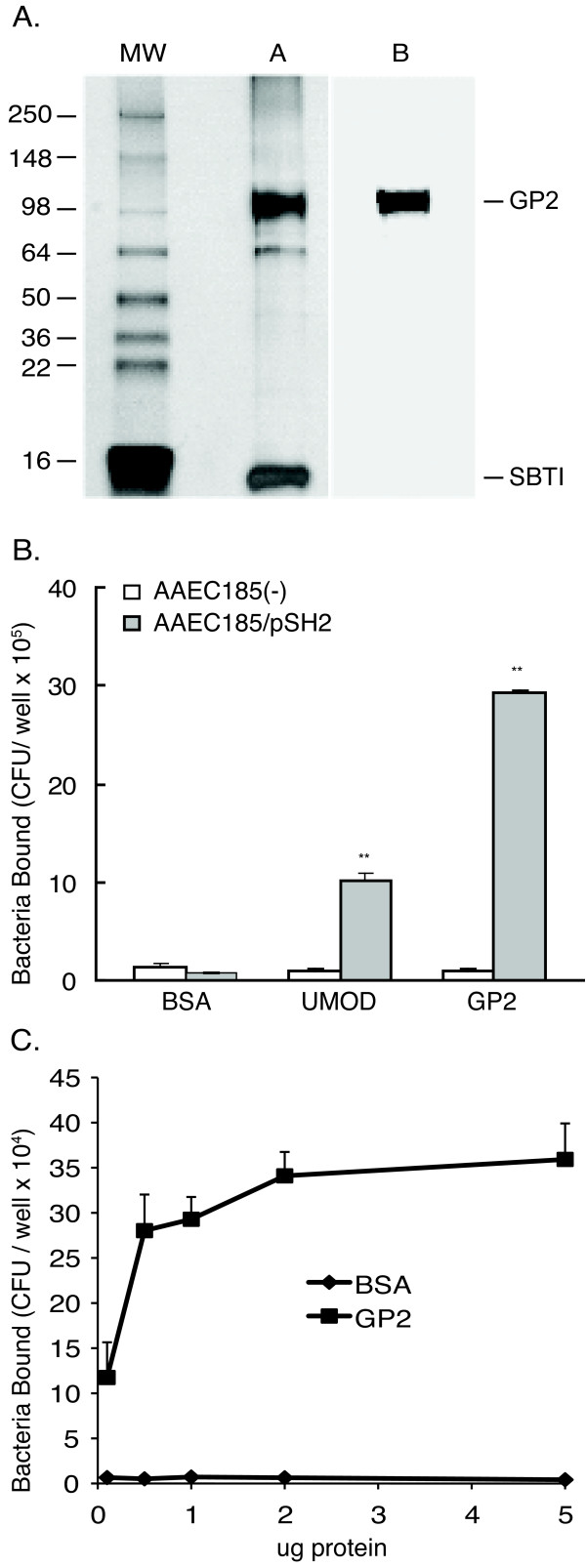
**GP2 binding to bacteria with Type 1 fimbria**. Silver stain of SDS-PAGE (a) and protein immunoblotting (b) of purified recombinant human GP2 protein. Protein immunoblotting was performed with rabbit anti-human GP2 antisera. SBTI, soybean trypsin inhibitor. (B) Binding assay of bacteria with (AAEC185/pSH2) and without (AAEC185) Type 1 fimbria performed in microtiter plates coated with 1 μg/well of GP2, uromodulin, or BSA protein. ** p < 0.01 compared with AAEC185. (C) Microtiter wells coated with GP2 or BSA proteins at concentrations of .1, .5, 1, 2 and 5 μg/ml, followed by incubation with AAEC185/pSH2 (1 × 10^8 ^CFU) bacteria that express Type 1 fimbria. All assays were performed in triplicate. The CFU/well are calculated as the mean ± SD (n = 3) and the error bar represents 1 SD.

Recombinant GP2 was purified from Chinese Hamster Ovary cells that were stably transfected with cDNA encoding the human GP2 gene. Subsequent analysis with gel electrophoresis revealed a dominant band at the expected size of 98 kDa and two small fragments at 64 kDa and 16 kDa (Figure 1A). Mass spectroscopy established that the 64 kDa was a degraded product of GP2 and the 16 kDa band is soybean trypsin inhibitor, which was added during the purification (data not shown). The purified GP2 retained antigenicity when probed with anti-human GP2 antibodies on immunoblots.

An *in vitro *binding assay was performed to test whether GP2 bound bacteria expressing type I fimbria that contained an intact FimH protein. Microtiter plates coated with purified GP2, human uromodulin, or BSA, were incubated with radiolabeled bacteria. Two bacterial strains were tested that differed only in their expression of Type 1 fimbriae. GP2 bound Type 1 fimbria expressing AAEC185/pSH2 bacteria and not the AAEC185 strain in which fimbriae are absent. Uromodulin showed binding properties similar to GP2. Type 1 fimbria expressing bacteria did not bind BSA (Figure 1B) or soybean trypsin inhibitor (data not shown).

Binding of Type 1 fimbria expressing bacteria (AAEC185/pSH2) was also examined at different plating concentrations for GP2 (Figure 1C). No significant bacterial binding to BSA was observed, whereas a dose dependent relationship was observed for GP2.

### Binding of Type 1 fimbria expressing bacteria to GP2 is specific

**Figure 2:**
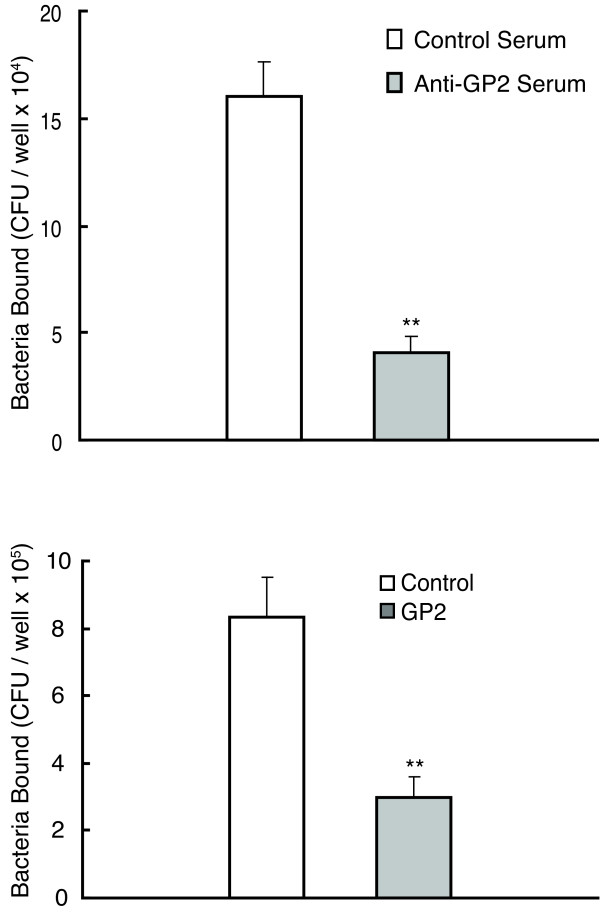
**Specificity of GP2 binding to bacteria with Type 1 fimbria**. (A) AAEC185/pSH2 bacteria binding assay with microtiter wells previously coated with 100 ng of GP2 protein followed by preincubation with either rabbit anti-human GP2 antiserum or control preimmune serum. (B) 5 μg of GP2 protein was preincubated with Type 1 fimbriated bacteria (AAEC185/pSH2) before application to the microtiter plates. Data are expressed as the mean CFU/well and the error bars equal 1SD. All assays were performed in triplicate. ** p < 0.01 compared with control preimmune serum (A) or without GP2 preincubation (B).

Two additional experiments were performed to evaluate the specificity of GP2 binding to FimH-expressing bacteria. First, GP2-coated microtiter plates were pre-incubated with rabbit anti-GP2 sera before bacterial binding was performed. The GP2 antisera inhibited AAEC185/pSH2 bacteria binding by 74% compared to normal sera derived from non-immunized rabbits (Figure 2A). Second, AAEC185/pSH2 bacteria were preincubated with purified recombinant human GP2 (50 μg/ml) before application to the GP2 coated plates (Figure 2B). GP2 preincubation inhibited bacterial binding by 64% compared to preincubation with BSA (p < 0.01).

### GP2 associated mannose is required for binding to FimH-expressing bacteria

**Figure 3:**
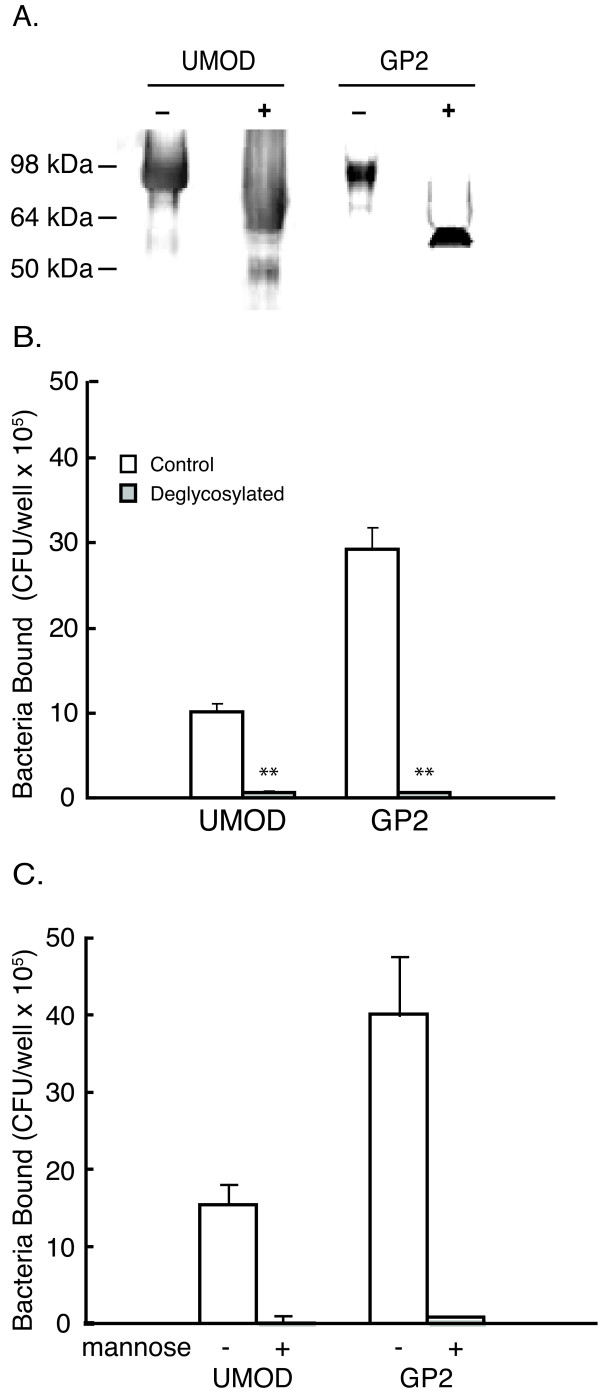
**GP2 glycosylation with mannose residues is required for bacteria binding**. (A) SDS-PAGE of GP2 and uromodulin (UMOD) proteins with (+) and without (-) deglycosylation with peptide:N-glycosidase F treatment. (B) Binding of Type 1 fimbriated bacteria (AAEC185/pSH2) to glycosylated and deglycosylated GP2 or UMOD. (C) AAEC185/pSH2 bacteria binding to GP2 after pre-incubation with 1% (v/v) α-D-mannose. Data are expressed as the mean CFU/well of triplicate samples and the error bar represents 1SD. ** p < 0.01 compared with untreated controls.

FimH containing Type I fimbria serve as a lectin that binds mannose residues [20,21]. Thus we examined whether GP2 glycosylation was similarly required for Type I fimbria mediated binding. Peptide:N-glycosidase F was used to removed asparagine-linked glycan chains from purified GP2 and uromodulin proteins before plating in the wells (Figure 3A). The binding of Type 1 fimbria expressing bacteria, AAEC185/pSH2, to deglycosylated GP2 was inhibited 98% compared to intact GP2 (Figure 3B). Similar results were obtained when uromodulin was treated in a similar manner.

To demonstrate mannose mediated GP2 binding to bacteria, AEC185/pSH2 bacteria were preincubated with 1% α-D-mannose before application to the GP2 coated microtiter plates. Mannose pretreatment of the bacteria decreased binding to GP2 or uromodulin by 98% (p < 0.01) (Figure 3C).

In the gastrointestinal tract, colonic epithelia contain receptors for both P and Type 1 fimbria [22]. P fimbria bind D-galactose residues. A binding assay was performed using the J96 bacteria strain, an uropathogenic strain that contains both Type I-FimH and P fimbria. Preincubation of J96 bacteria with 1% α-D-mannose resulted in an 86% decrease in J96 binding to GP2, indicating that binding is mediated mainly by Type I fimbria (data not shown).

### GP2 Binds to M_1_H and M_1_L variants of FimH

**Figure 4:**
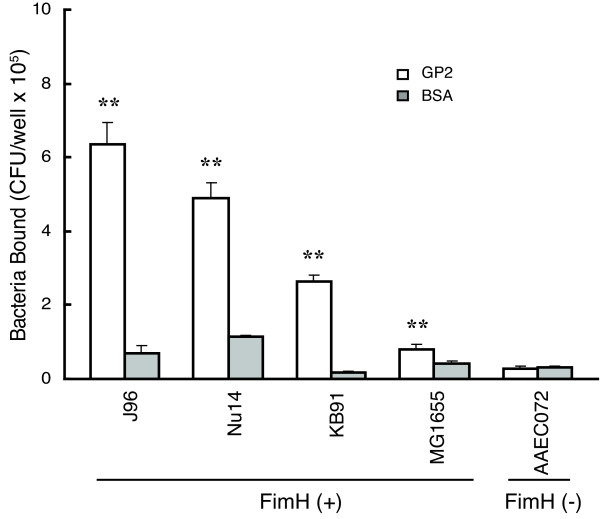
**GP2 binding to bacteria with M_1_H and M_1_L Type 1 fimbriae**. Different bacteria strains were tested for the ability to bind GP2 in the microtiter well binding assay. 1 × 10^8 ^CFU/well bacteria were used for each assay. FimH designates bacteria with or without FimH containing Type 1 fimbria. Bound bacteria were expressed as the mean CFU/well and the error bar represents 1SD (n = 3). ** p < 0.01 compared to BSA.

The FimH subunit of Type 1 fimbriae in uropathogenic and enteropathogenic strains of E. coli are known to differ in their respective affinities for mannose residues. FimH in uropathogenic E. coli strains exhibit high affinity binding to mono-mannose residues, whereas enteropathogenic FimH possesses low affinity mono-mannose binding. Variations in the FimH sequence account for the different affinities [23,24]. GP2 did not bind AAEC072 bacteria in which the FimH gene was deleted, or MG1655 bacteria in which Type 1 fimbria is largely phased off (Figure 4). GP2 bound KB19, a recombinant strain that expresses the low affinity M_1_L variant of FimH isolated from the intestinal F-18 *E. coli *strain [25]. GP2 also bound the Nu14 *E. coli *strain that expresses the high affinity M_1_H variant of FimH. Last, GP2 also bound the uropathogenic *E. coli *strain, J96, which expresses the M_1_H variant of FimH and also P fimbriae.

## Discussion and Conclusion

Among bacterially expressed adhesins, Type I fimbria is the most common among the entire family of *Enterobacteriaceae*. Type I fimbria is also the most common adhesin expressed by *E. coli *in the gastrointestinal and urinary tracts. About 60% (30–100%) of fecal isolates express Type 1 fimbria whereas only 20% (7–52%) express P fimbria [26]. Among patients septic from ascending cholangitis, *E. coli *was cultured from 76% of the episodes, and FimH was expressed by 88% of the *E. coli *isolates [27]. Thus *E. coli *with Type I fimbria represent a major pathogen in both the gastrointestinal and urogenital tracts.

Variations in the adhesive properties of Type I fimbriae are characterized by either high (M_1_H) or low (M_1_L) affinity mannose binding, which may specify differences in host cell binding [24,25]. Eighty percent of intestinal isolates bind monomannosyl residues with low affinity (M_1_L phenotype) and 70% of urinary isolates bind monomannosyl residues with high affinity (M_1_H phenotype) [25]; with differences in affinity measured as high as 15-fold. In this study, we demonstrate that GP2 is capable of binding both M_1_H and M_1_L variants of FimH.

Uromodulin is known to self assemble into a macromolecular structure and represents the major constituent of renal casts. Once secreted, GP2 has also been described to assemble into a fibrillar network [28]. Both GP2 and uromodulin contain a ZP domain that is responsible for its polymerization in the extracellular space [29-31]. Thus similar to uromodulin in the kidney, GP2 binding to Type I fimbriae may serve as a physical barrier and as a molecular decoy for bacterial adhesion. Although the common bile duct and pancreatic duct share a common exit to the intestine, ascending infections of the pancreatic duct have not been reported in the literature and clearly are not commonly observed in the clinical setting. In contrast, ascending biliary tract infections secondary to bacteria derived from the intestine are relatively common. Even when the pancreatic duct is obstructed with protein or stones in chronic pancreatitis, ascending infections are not reported. It is of interest that GP2 comprises most of the protein precipitate present in the duct of patients afflicted with chronic pancreatitis, and thus may serve a protective role against infection [32].

Uromodulin's role in host defense has been supported in an animal model in which *UMOD null *mice expressed more persistent infections when uropathogenic bacteria were introduced into the urinary bladder [14]. Similar experiments have yet to be successful in *GP2 null *mice, which may be secondary to several factors. First, an animal model for ascending pancreatic duct infections has not been established. Second, almost all investigative *E. coli *strains are derived from the intestine or urogenital tract. As observed in the intestine and urogenital tract, numerous strains show sequence variation in FimH, which represents an adaptation to a different host environment. Because ascending infections of the pancreatic duct have not been observed, bacterial strains have not been isolated from the pancreatic duct and thus may compromise current efforts at defining *in vivo *GP2's role in host defense. Future studies will require the isolation of bacterial strains that thrive in the pancreatic duct. Such bacteria may include those predisposed to the biliary tract. Overall, the similarities between GP2 and uromodulin in biochemical structure and binding to Type 1 fimbriae support a role for GP2 in host defense.

Recent studies have also demonstrated GP2 expression by M cells in follicle-associated epithelium of intestinal Peyer's patches [33]. Whether GP2 binding to bacteria expressing Type 1 fimbria serves a role in the intestinal Peyer's patch represents a promising area for further investigation.

## Abbreviations

*E. coli*: Escherichia coli; SDS: sodium dodecyl sulfate; BSA: bovine serum albumin; SD: Standard deviation; CFU: colony forming units.

## Competing interests

The authors declare that they have no competing interests.

## Authors' contributions

SY participated in the experimental design, conduct of the experiments, data analysis, and drafting of the manuscript. AWL conceived the project and participated in the experimental design, data analysis, and drafting of the manuscript. All authors read and approved of the final manuscript.

## Pre-publication history

The pre-publication history for this paper can be accessed here:

http://www.biomedcentral.com/1471-230X/9/58/prepub
